# Sequential transfection of RUNX2/SP7 and ATF4 coated onto dexamethasone-loaded nanospheres enhances osteogenesis

**DOI:** 10.1038/s41598-018-19824-x

**Published:** 2018-01-23

**Authors:** Hye Jin Kim, Ji Sun Park, Se Won Yi, Hyun Jyung Oh, Jae-Hwan Kim, Keun-Hong Park

**Affiliations:** Department of Biomedical Science, College of Life Science, CHA University, 6F, CHA Biocomplex, 689 Sampyeong-dong Bundang-gu, Seongnam-si, 134-88 Korea

**Keywords:** Stem-cell differentiation, Stem-cell research

## Abstract

The timing of gene transfection greatly influences stem cell differentiation. Sequential transfection is crucial for regulation of cell behavior. When transfected several days after differentiation initiation, genes expressed at the late stage of differentiation can regulate cell behaviors and functions. To determine the optimal timing of key gene delivery, we sequentially transfected human mesenchymal stem cells (hMSCs). This method can easily control osteogenesis of stem cells. hMSCs were first transfected with RUNX2 and SP7 using poly(lactic-co-glycolic acid) nanoparticles to induce osteogenesis, and then with ATF4 after 5, 7, and 14 days. Prior to transfecting hMSCs with all three genes, each gene was individually transfected and its expression was monitored. Transfection of these genes was confirmed by RT-PCR, Western blotting, and confocal microscopy. The pDNAs entered the nuclei of hMSCs, and RUNX2 and SP7 proteins were translated and triggered osteogenesis. Second, the ATF4 gene was delivered when cells were at the pre-osteoblasts stage. To induce the osteogenesis of hMSCs, the optimal timing of ATF4 gene delivery was 14 days after RUNX2/SP7 transfection. Experiments in 2- and 3-dimensional culture systems confirmed that transfection of ATF4 at 14 days after RUNX2/SP7 promoted osteogenic differentiation of hMSCs.

## Introduction

Stem cell differentiation depends on several conditions, related to processes both upstream and downstream of transcriptional factors^1–3^. During development, several signaling factors participate in chondrogenesis, osteogenesis, and adipogenesis^4–6^. The *SOX9* gene is a key player in chondrogenic differentiation of stem cells^7^. *SOX9* expression in stem cells increases the level of the extracellular matrix (ECM) protein aggrecan, which increases the levels of collagen type II (COL2A1) and other chondrogenic factors^8–10^. C/EBPα and PPARγ are master regulators of adipogenesis and part of a highly ordered network of transcription factors^11^. Adipogenesis of stem cells requires C/EBPβ-mediated expression of PPARγ^12^. The Smad and non-Smad pathways play important roles in osteogenesis of stem cells^13,14^. Induction of the Runt-related transcription factor 2 (RUNX2), osterix, and ATF4 genes can control the Smad-dependent pathway to drive osteogenesis of stem cells^15–18^. Combinations of exogenous genes are necessary to modulate the Smad-dependent pathway and thereby induce osteogenesis of stem cells^19^. The timing of exogenous gene delivery can control stem cell differentiation. To activate the upstream transcriptional signals such as RUNX2 and SP7 target genes, their expression induces differentiation of stem cells into pre-osteoblasts^20,21^. RUNX2 protein plays an important role during osteogenic differentiation of stem cells^22–25^. Although the RUNX2 gene induces osteogenesis of stem cells, the final stage is dependent on the expression of other genes. Delivery of the final-stage gene ATF4 can promote osteogenesis of stem cells. The early osteogenesis-related genes RUNX2 and SP7 triggered conversion of stem cells into pre-osteoblasts and then subsequent delivery of the ATF4 gene then stimulated further osteogenesis. Sequential transfection of RUNX2/SP7 followed by ATF4 enhanced osteogenesis of hMSCs.

## Results

### Vector confirmation and characterization of DNPsP coated with pDNAs

**Figure 1:**
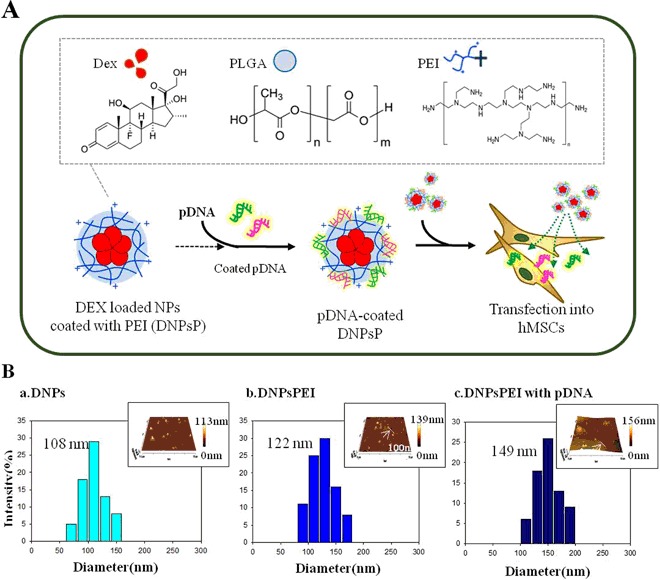
Characterization of dexamethasone-loaded nanoparticles (DNPs). (**A**) Schematic illustration of DNPs formation. (**B**) Size and distributiondata abtained by dynamic light scattering (DLS) and atomic force microscopy (AFM) scanning. (a) DNPsP, (b) DNPsP with pDNA.

**Figure 2:**
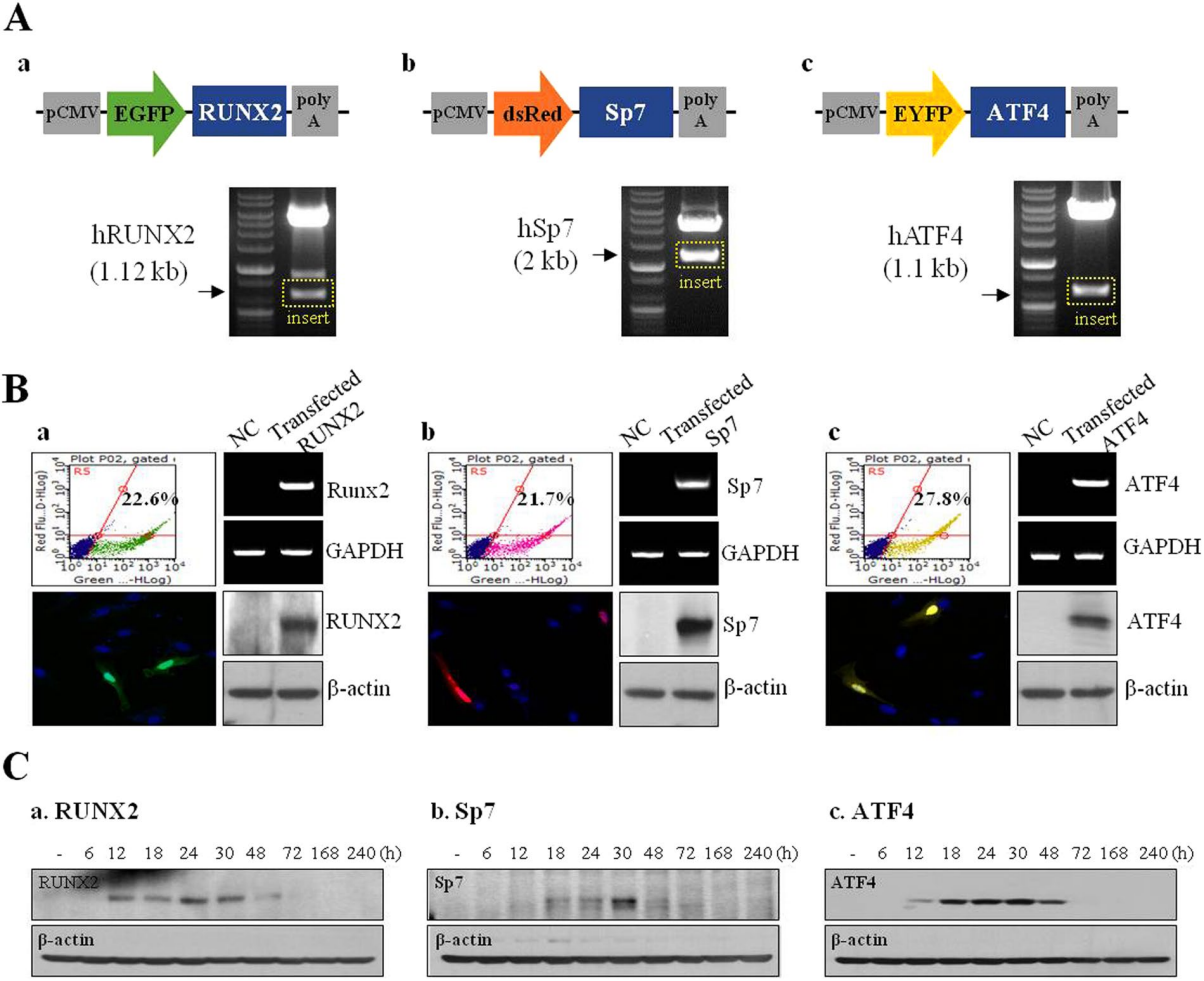
Fabrication and characterization of pDNAs harboring the RUNX2, SP7, and ATF4 genes. (**A**) Maps of plasmids harboring (a) EGFP-RUNX2, (b) dsRed-SP7, and (c) EYFP-ATF4. (**B**) Effects of exogenous gene expression on mRNA and protein levels of (a) RUNX2, (b) SP7, and (c) ATF4 in hMSCs detected by FACS, RT-PCR, confocal laser microscopy, and Western blot analyses. (**C**) Production of (a) RUNX2, (b) SP7, and (c) ATF4 proteins over time following transfection of the corresponding gene.

Figure 1A shows aschematic illustration for the nanoparticles formation of DNPs. We first characterized these pDNA-coated non-toxic NPs by performing, dynamic light scattering (DLS) and atomic force microscopy (AFM) experiments (Fig. 1B). The size of DNPs, DNPsPEI, and DNPsPEI with pDNA were 108 nm, 122 nm, and 149 nm, respectively. Complexation with pDNA changed the size and surface charge of NPs. We than are second characterized the expression vectors harboring EGFP-RUNX2, dsRed-SP7, and EYFP-ATF4. The vector maps are provided in Fig. 2A. These vectors were confirmed by nucleotide sequencing, restriction enzyme digestion, and gel electrophoresis (Fig. 2A,a–c). Transfection of hMSCs with DNPsP coated with pDNAs harboring the RUNX2, SP7, and ATF4 genes were tested (Fig. 2B). To monitor their internalization, the genes were tagged with a fluorescent marker (EGFP, dsRed, or EYFP). Fluorescence activated cell sorting (FACS) analysis demonstrated that 22.6%, 21.7%, and 27.8% of hMSCs were transfected with EGFP-RUNX2, dsRed-SP7, and EYFP-ATF4, respectively.

Gene and protein expression of RUNX2, SP7, and ATF4 was detected by RT-PCR and Western blot analyses, respectively. This demonstrated that the internalized pDNAs easily entered the nucleus, and then the mRNAs were expressed and translated into proteins.

However, RUNX2, SP7, and ATF4 expression was not detected in non-transfected cells. Transfected cells were imaged by confocal laser microscopy. Green, red, and yellow fluorescence signals were detected when the corresponding genes were transfected. This demonstrates that the transfected genes were translated into proteins that were localized in the nucleus and cytosol. We determined the protein expression levels of RUNX2, SP7, and ATF4 at various times after transfection by performing Western blot analysis (Fig. 2C). All three proteins were highly expressed at 30 h and their expression was maintained for 48 h. This demonstrates that the internalized genes were active for more than 48 h (Fig. 2C,a–c).

The full vector maps are provided in Fig. S1A. 293 T cells were also successfully transfected with the pDNAs (Fig. S2B). The pDNAs easily entered both hMSCs and 293T cells and the specific proteins were expressed.

**Figure 3:**
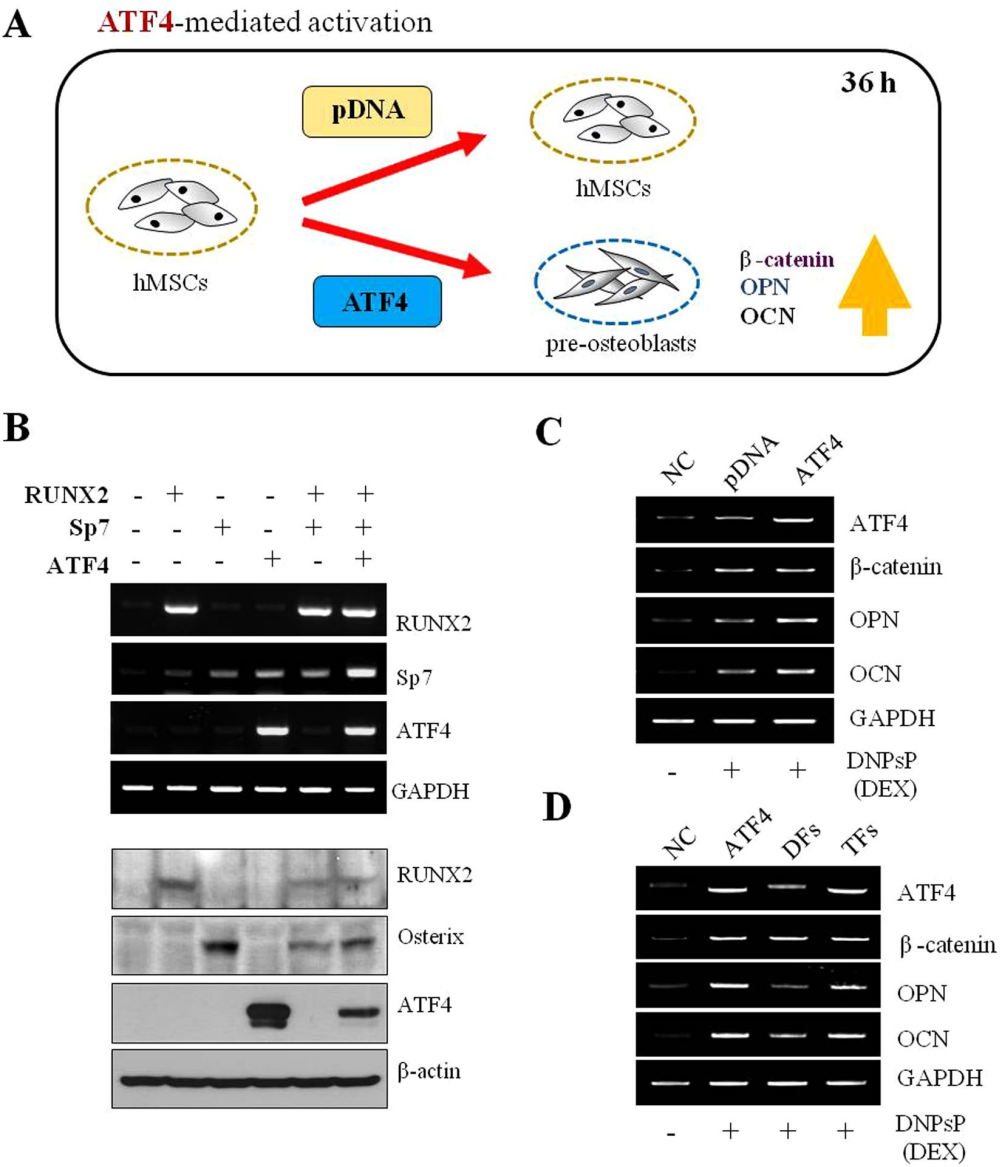
RT-PCR analysis of hMSCs treated with DNPsP coated with pDNA or pATF4. (**A**) Schematic illustration of the effects of treatment with DNPsP coated with pDNA orpATF4 on hMSCs. (**B**) RT-PCR and Western blot analysis of mRNA and protein levels in hMSCs transfected with one, two, and three genes. (**C**) RT-PCR analysis of mRNA expression levels of ATF4, CTNNB1, OPN, and OCN in hMSCs treated with DNPsP coated with pDNA or pATF4. (**D**) RT-PCR analysis of mRNA expression levels of ATF4, CTNNB1, OPN, and OCN in hMSCs treated with DNPsP coated with pDNAs harboring ATF4, RUNX2 plus SP7, or all three genes.

This demonstrates that non-toxic NPs were easily complexed with the fabricated pDNAs. The mRNA and protein levels of RUNX2, SP7, and ATF4 were determined by RT-PCR and Western blot analyses, respectively, in hMSCs transfected with the three factors, either separately or in combination (Fig. 3B). hMSCs transfected with each pDNA individually demonstrated mRNA expression of the corresponding gene. hMSCs co-transfected with RUNX2 and SP7(DFs) demonstrated mRNA expression of both genes. Finally, hMSCs co-transfected with RUNX2, SP7, and ATF4(TFs) demonstrated mRNA expression of all three genes, showing that they were internalized and entered the nucleus (Fig. 3B). To determine whether the internalized genes were translated into proteins, Western blot analysis was performed (Fig. 3B). In line with the RT-PCR findings, RUNX2, SP7, and/or ATF4 proteins were detected following the transfection of one, two, or three factors. hMSCs transfected with only RUNX2 or SP7 expressed the corresponding protein, hMSCs co-transfected with RUNX2 and SP7 expressed both proteins, and hMSCs co-transfected with RUNX2, SP7, and ATF4(TFs) expressed all three proteins. These results indicated that the internalized gene scanenter the nuclei of the stem cells and be translated into proteins.

### Evaluation of cytotoxicity of DNPsP coated with pDNA

To determine the cytotoxic effects of pDNA-coated NPs, live and dead cells were detected using Cell Counting Kit-8 (CCK-8) and the live/dead imaging assay (Fig. S1A and B). These results indicated that NPs coated with pDNAs harboring one, two, and three genes did not induce cell death. Thus, NPs complexed with pDNAs are safe and effective gene delivery carriers. To elucidate the locations of the exogenous genes, we monitored protein expression and fluorescence. RUNX2, SP7, and ATF4 were present in both the cytosol and nucleus (Fig. S2A and B).

### Studies of osteogenesis according to the timing of pATF4-coated DNPsP transfection

Osteogenic differentiation of hMSCs transfected with ATF4 only, RUNX2 plus SP7 (DFs), and all three factors (TFs) was tested (Fig. 3). For transfection of all three genes, the ATF4 gene was delivered into hMSCs that were treated with RUNX2 and SP7 genes before 36 h ago prior to the transfection. The procedure is illustrated in Fig. 3A. The mRNA levels of osteogenic markers differed according to whether the ATF4 gene was transected. ATF4, β-catenin, OPN, and OCN wereclearly expressed in the hMSCs transfected with pATF4 (Fig. 3C).

The effects of transfection of ATF4, DFs, and TFs on theosteogenesis of hMSCs were investigated by performing RT-PCR (Fig. 3D). mRNA expression of ATF4, CTNNB1, OPN, and OCN differed depending on which genes were transfected. Expression of these marker genes was higher in hMSCs transfected with TFs than in the hMSCs transfected with DFs and non-transfected control hMSCs. This demonstrates that transfection of ATF4 has a better potential to induce osteogenesis of hMSCs than transfection of two factors (DFs).

### Studies of osteogenesis by transfection timing of ATF4 gene

**Figure 4:**
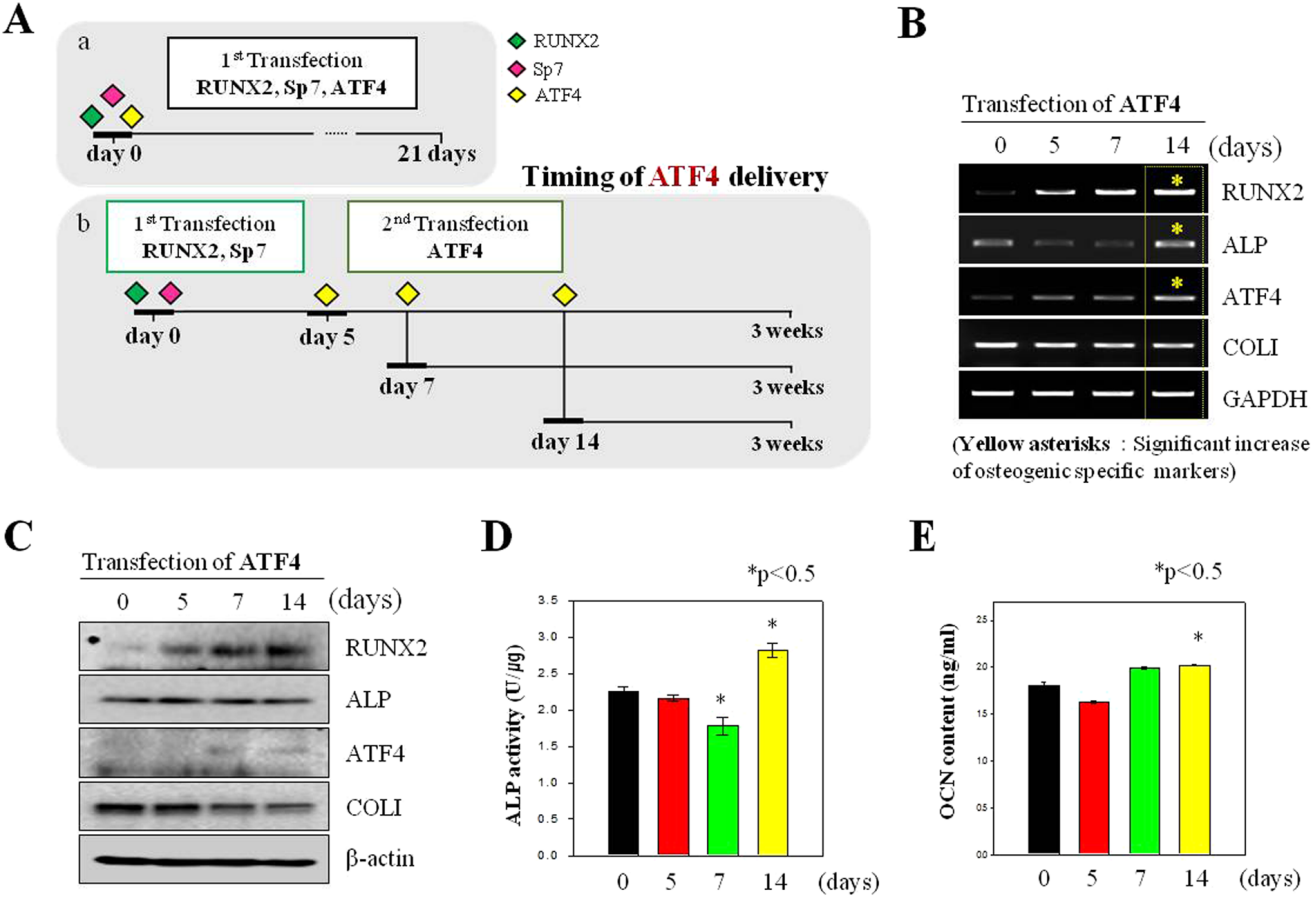
Transfection of hMSCs with RUNX2/SP7 followed by ATF4. (**A**) Schematic illustration of transfection of (a) all three genes at the same time and (b) ATF4 at 5, 7, and 14 days after RUNX2/SP7. (**B** and **C**): (**B**) RT-PCR and (**C**) Western blot analyses of the mRNA and protein expression levels of RUNX2, ALP, ATF4, and COL1A2 in hMSCs transfected with ATF4 at 5, 7, and 14 days after RUNX2/SP7. (**D** and **E**): Quantified levels of (**D**) ALP activity and (**E**) OCN expression in hMSCs transfected with ATF4 at 5, 7, and 14 days after RUNX2/SP7. *P < 0.05, **P < 0.01.

To optimize the triple gene delivery method, we modified the timing of the ATF4 delivery (Fig. 4). ATF4 is involved in the final stage of osteogenesis and has potential actions when internalized by stem cells. We transfected hMSCs with ATF4 5, 7, and 14 days after RUNX2/SP7 and then cultured these cells to investigate osteogenesis (Fig. 4A). Alternatively, all three genes were transfected at the same point in time. mRNA and protein levels of the osteogenesis markers were investigated (Fig. 4B and C). When all three genes were delivered at the same time point, the mRNA levels of RUNX2, ALP, ATF4, and COL1A2 were slightly lower than those in the other samples. The mRNA and protein levels of RUNX2 and ATF4 were higher when ATF4 was transfected at 5, 7, or 14 days after RUNX2/SP7 than when all three genes were delivered together. Specifically, the mRNA and protein levels of RUNX2, ALP, ATF4, and COL1A2 were high when ATF4 was delivered 14 days after RUNX2/SP7. Thus, this showed the optimal timing of ATF4 delivery to induce osteogenic differentiation of hMSCs. The protein expression levels of ALP and OCN were quantified to determine theoptimal timing of ATF4 transfection (Fig. 4D and E). ALP and OCN protein expression differed according to the timing of the ATF4 delivery. Although protein expression of ALP and OCN did not differ between the cells transfected with ATF4 after 5 or 7 days, it was higher in cells transfected with ATF4 after 14 days. hMSCs transfected with ATF4 after 5, 7, and 14 days were compared by performing trichrome staining (Fig. S3A). The staining was most intense in the hMSCs transfected with ATF4 after 14 days (Fig. S3A,a–d). Moreover, immunofluorescence staining of COL1A2 (green) was detected in these cells (Fig. S3B,a–d). All osteogenesis-related proteins were highly expressed in the hMSCs transfected with ATF4 after 14 days. hMSCs co-transfected with RUNX2 plus SP7 were cultured for 14 days prior to ATF4 transfection; however, these two genes were insufficient to drive osteogenesis of hMSCs. To evaluate the necessity of the ATF4 gene for osteogenesis, the MOCK and ATF4 genes were transfected.

**Figure 5:**
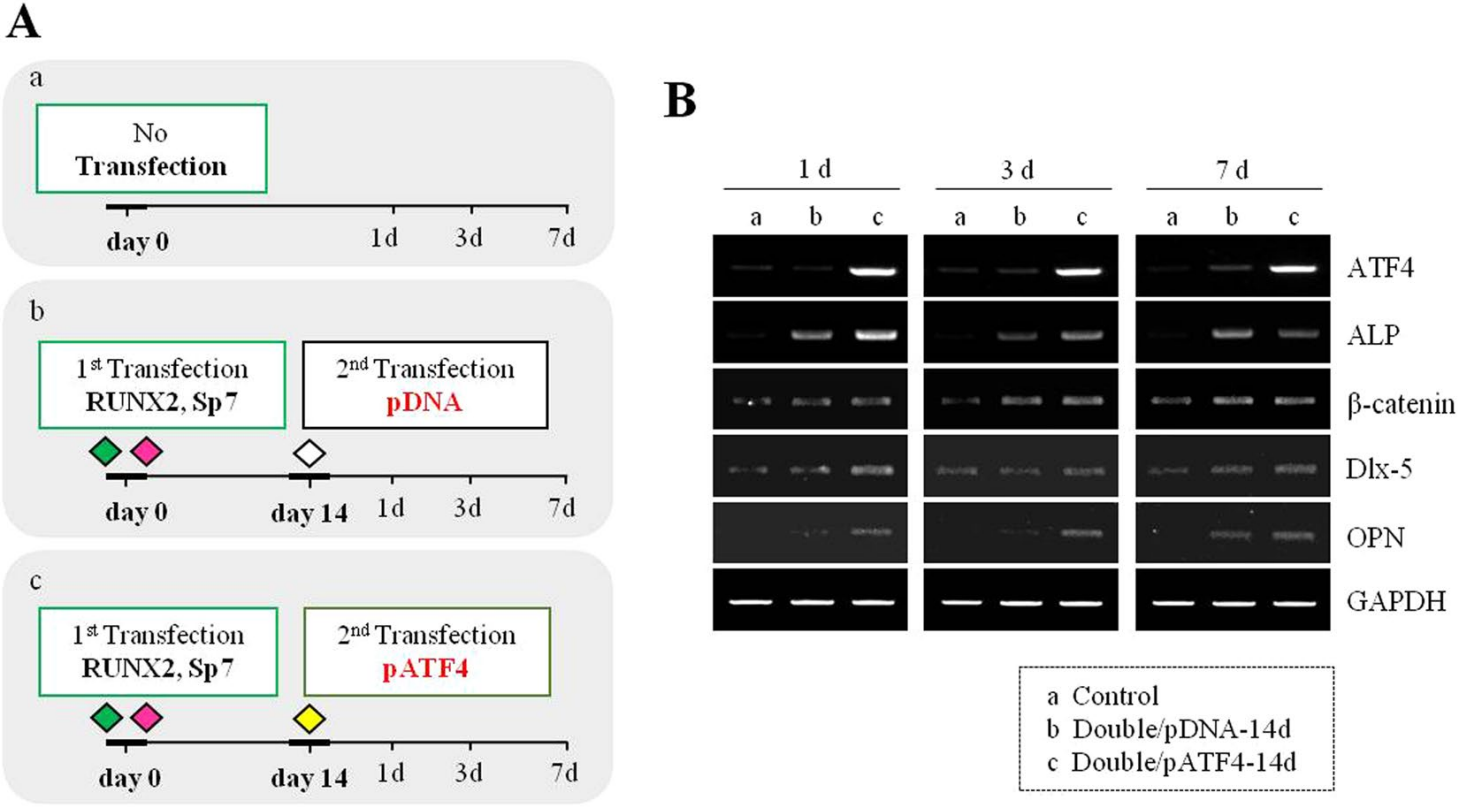
Gene expression following transfection of hMSCs with DNPsP coated with pDNA or pATF4. (**A**) Schematic illustration of the schedule used to determine the genes expressed upon ATF4 delivery. (a) Negative control, (b) transfection of pDNA at 14 days after RUNX2/SP7, and (c) transfection of pATF4 at 14 days after RUNX2/SP7. (**B**) RT-PCR analysis of the mRNA expression levels of ATF4, ALP, CTNNB1, Dlx5, and OPN at 1, 3, and 7 days after transfection of hMSCs with DNPsP coated with pDNA or pATF4.

The experimental procedure is illustrated in Fig. 5A. hMSCs were transfected with the MOCK or ATF4 gene at 14 days after RUNX2/SP7 (Fig. 5B). At 1, 3, and 7 days after transfection of the ATF4 or MOCK gene, we compared the osteogenic differentiation of hMSCs. Expression of ALP, CTNNB1, DLX5, OPN, and ATF4 were higher in the hMSCs transfected with ATF4 than in the other groups (Fig. 5B). ATF4 gene expression was maintained. This demonstrates that ATF4, a gene related to the final stage of osteogenesis, helps to convert hMSCs into osteoblasts. The gene and protein levels of ATF4 and COL1A2 were high in the hMSCs transfected with ATF4 14 days after RUNX2/SP7 and cultured for 1, 3, 5, and 7 days (Fig. S4). COL1A2 gene and protein expression was highest in the hMSCs cultured for 7 days after ATF4 transfection (Fig. S4A).

### Effects of the delivered genes on osteogenesis in 2D culture system

**Figure 6:**
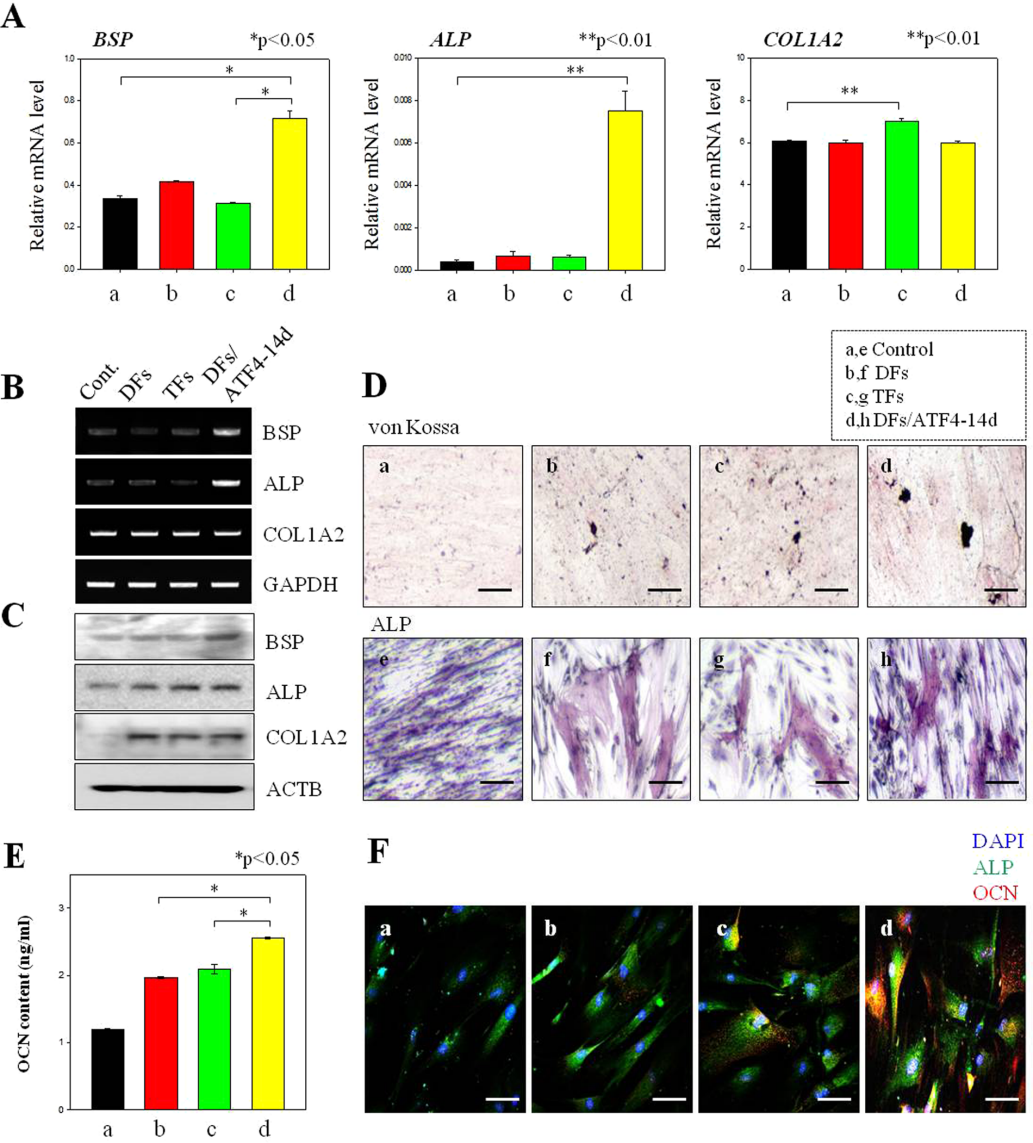
Osteogenesis of hMSCs transfected with two and three genes in a 2D culture system. (**A)** mRNA expression levels of osteogenesis-related markers in hMSCs transfected with two and three genes. (**B**) Protein expression levels of osteogenesis-related markers in hMSCs transfected with one, two, and three genes. (**C**) Histological analysis of osteogenesis in hMSCs transfected with one, two, and three genes. (**D**) Quantified levels of OCN in hMSCs transfected with one, two, and three genes. *P < 0.05. (**E**) Immunofluorescence analysis of (a) control hMSCs and those transfected with (b) two genes, (c) all three genes at the same time point, and (d) ATF4 at 14 days after RUNX2/SP7 (blue, DAPI; green, ALP; and red, OCN). Scale bar, 100 μm.

To confirm the effects of the delivered genes on osteogenesis of hMSCs, the cells were transfected in various ways, grown for 4 weeks in 2D cultures, and then subjected to q-PCR, RT- PCR, Western blot, and histological analyses (Fig. 6). Specifically, hMSCs were transfected with RUNX2 plus SP7 (DFs), with TFs at the same point in time, or with ATF4 14 days after RUNX2/SP7 (DFs/ATF4-14d). RT-PCR and qPCR analysis of BSP, ALP, and COL1A2, which are marker genes of mature osteoblasts, demonstrated that sequential transfection was the best option (Fig. 6A and B).

BSP, ALP, and COL1A2 proteins were detected inthe hMSCs transfected with DFs or TFs, but not in the non-transfected hMSCs (Fig. 6C). However, there were some differences between the hMSCs transfected with two genes and those transfected with three genes. Osteogenesis was more effectively induced by the delivery of TFs. These results indicate that the timing of gene delivery can greatly influence stem cell differentiation.

Analysis of specific markers demonstrated that transfected hMSCs started to produce ECM proteins. For formation of bone tissues, mineralization and calcification were potential roles in the microenvironment. To detect mineralization and the ALP content of the hMSCs transfected with several genes, we performed von Kossa (Fig. 6D,a–d) and ALP (Fig. 6D,e–h) staining after 4 weeks in culture. No black or red labeling, indicative of mineralization and calcification, was detected in cultured the hMSCs transfected with two genes, with TFs at the same point in time, and with ATF4 after 14 days (DFs DFs/ATF4-14d). However, we observed mineralization in some hMSCs areas (Fig. 6D,d). Although the area of staining was small, this demonstrates the differentiation of the hMSCs into mature osteoblasts. To quantify this differentiation, OCN production, which represents the conversion of hMSCs into osteoblasts (Fig. 6E), was measured OCN production differed based on the treatment of the hMSCs. OCN production was higher in the hMSCs transfected with all three genes than in the control hMSCs and in those transfected with only DFs (Fig. 6E,c and d). Moreover, OCN production was higher in the hMSCs transfected with DFs/ATF4-14d than in the hMSCs transfected with TFs at the same point in time. This demonstrates that delivery of DFs/ATF4-14d has better potential to induce osteogenesis than the other delivery protocols.

We performed immunofluorescence analysis to confirm the osteogenic differentiation of hMSCs transfected with several genes (Fig. 6F). Protein markers of mature osteoblasts were expressed in the hMSCs transected with several genes. hMSCs transfected with ATF4 after DFs demonstrated vivid green and red staining, representing ALP and OCN proteins, respectively. Although green and red staining was also observed in the hMSCs transfected with all three genes at the same point in time, it was not as intense. This indicates that the hMSCs transfected with DFs/ATF4-14d produce higher levels of proteins related tomature osteoblasts.

### Effects of the delivered genes on osteogenesis in 3D culture system

**Figure 7:**
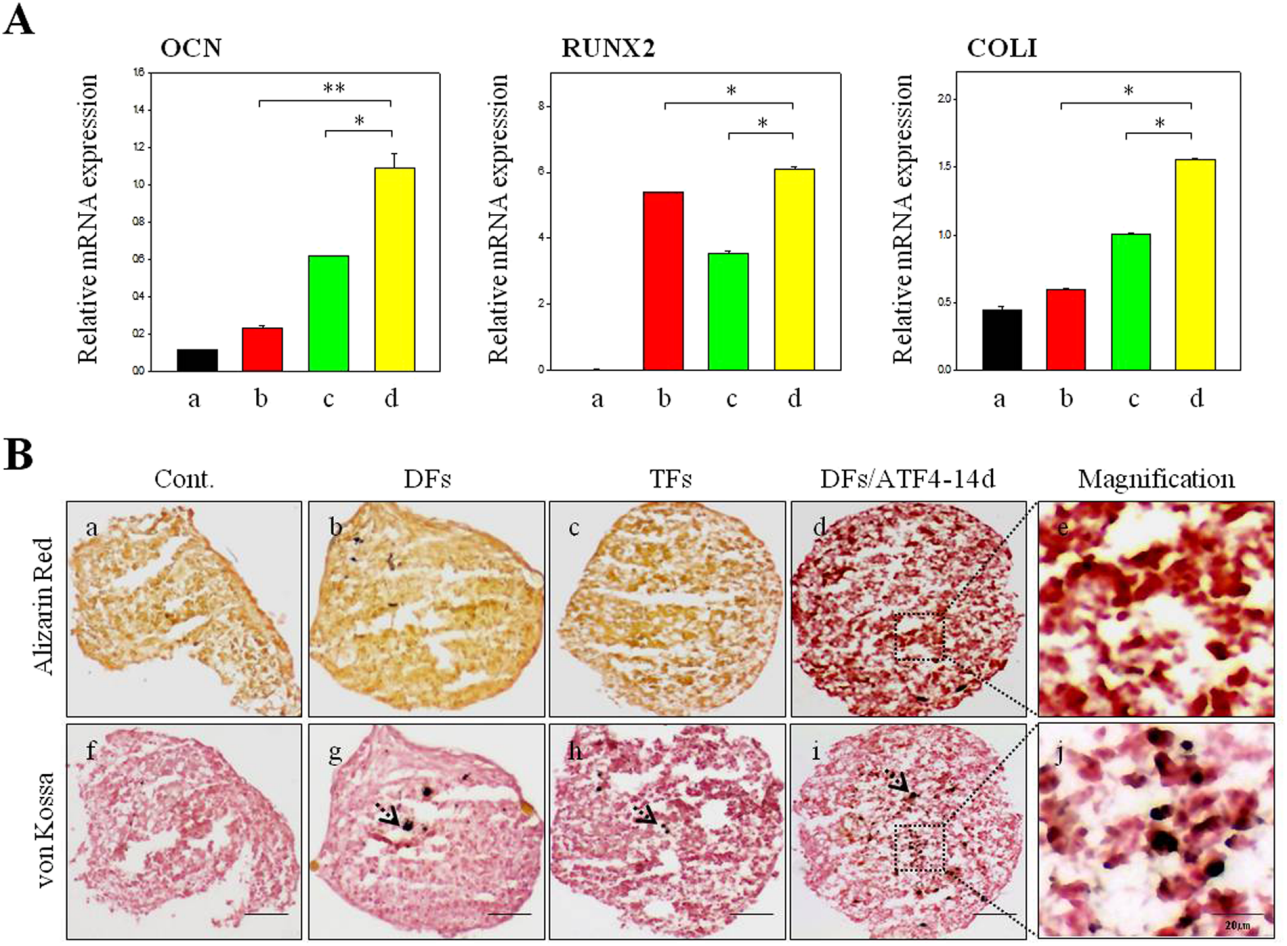
Osteogenesis of hMSCs transfected with two and three genes in a 3D culture system. (**A**) qPCR analysis of OCN, RUNX2, and COL1A2 mRNA levels in hMSCs transfected with two and three genes. *P < 0.05, **P < 0.01. (**B**) Histological analysis of osteogenesis. (a–e) Alizarin Red S staining and (f–j) von Kossa staining. (a and f) control; (b and g) hMSCs transfected with two genes; (c and h) hMSCs transfected with three genes at the same time point; (d and i) hMSCs transfected with ATF4 at 14 days after RUNX2/SP7; (e and j) magnifications of the images shown in panels (d and i). Scale bar, 200 μm.

hMSCs transfected in the various ways were grown in a 3D culture system to confirm their osteogenic differentiation (Fig. 7). The expression levels of the mature osteoblast-related genes were measured by qPCR to determine the effect of sequential transfection (Fig. 7A). The observations of the hMSCs transfected with ATF4 after DFs in the 3D culture system were identical to those in the 2D culture system. Expression of the OCN, RUNX2, and COL1A2 marker genes was higher in these hMSCs than in the other groups. This demonstrates that the timing of transfection can influence stem cell differentiation. The expression levels of these marker genes were quantified (Fig. 7A). Similar to the PCR results, their expression levels were much higher in the sequentially transfected hMSCs than in the other groups.

Specifically, their expression levels marker genes in hMSCs transfected with ATF4 after RUNX2/SP7 were twice as high as those in marker genes hMSCs transfected with TFs and five times higher than in the hMSCs transfected with DFs. This demonstrates that sequential transfection promoted osteogenic differentiation of hMSCs. The extent of osteogenesis in the various groups of hMSCs was determined by staining with Alizarin Red S and von Kossa to detect calcium deposition and mineralization, respectively (Fig. 7B). For Alizarin Red S staining, red spots were observed close to the sequentially transfected hMSCs, representing calcium deposition (Fig. 7B,d and e). In the case of von Kossa staining, black spots near the hMSCs were detected in all groups except the control group (Fig. 7B,g–j).

**Figure 8:**
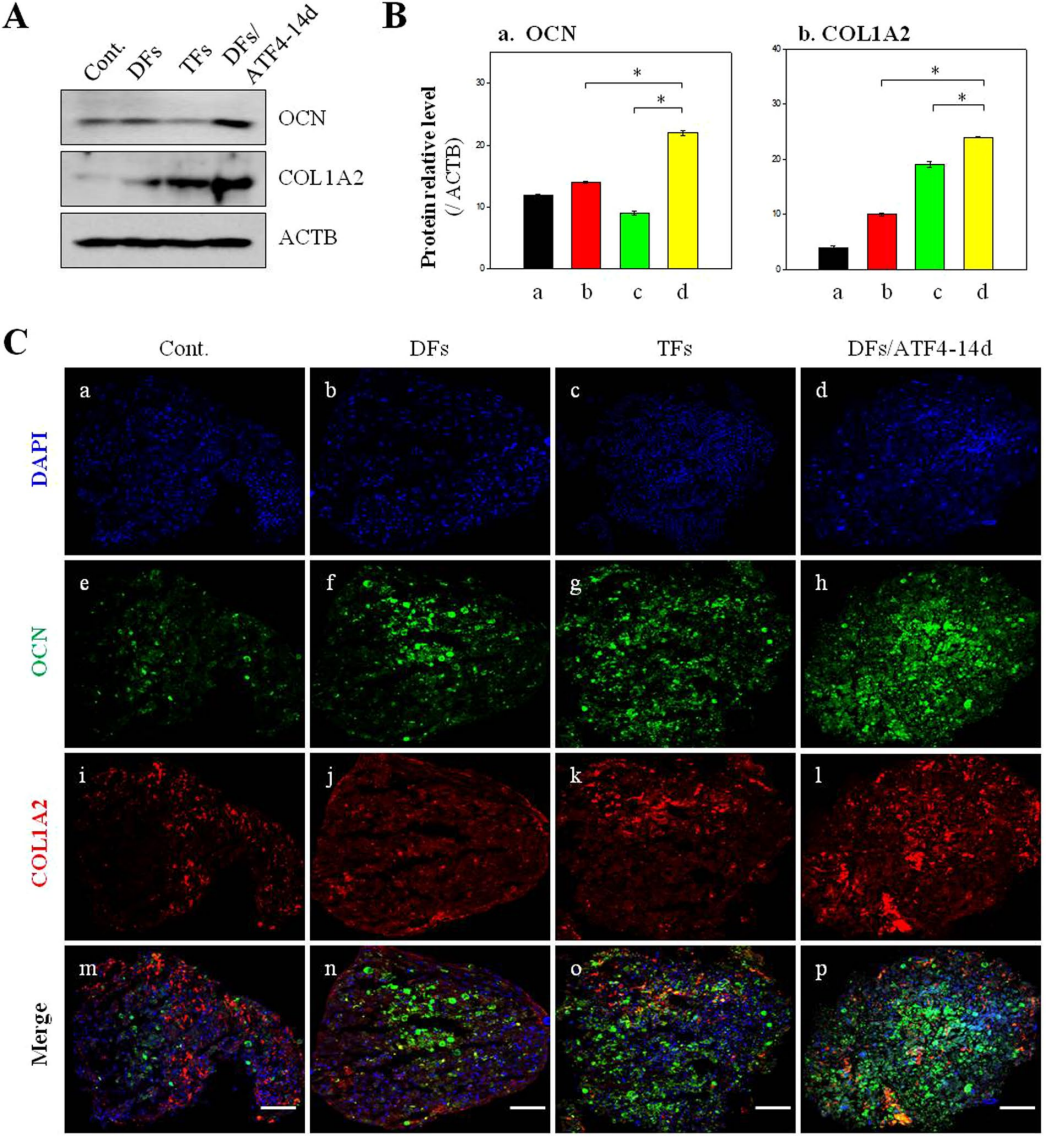
Osteogenesis of hMSCs transfected with two and three genes determined by Western blot analysis, quantification of protein levels, and immunohistology. (**A**) Western blot analysis of hMSCs transfected with two and three genes. (**B**) Quantification of (a) OCN and (b) COL1A2 protein levels in hMSCs transfected with two and three genes. *P < 0.05. (**C**) Immunohistological analysis of osteogenesis. (a–d) DAPI staining; (e–h) OCN staining; (i–l) COL1A2 staining; and (m–p) merged images. Scale bar, 200 μm.

More black spots were observed in the sequentially transfected hMSCs than in the hMSCs transfected with DFs and hMSCs transfected with TFs (Fig. 7B,i and j). This demonstrates that hMSCs transfected with ATF4 after RUNX2/SP7 can differentiate into osteoblasts. We determined the protein levels of OCN, and COL1A2 by Western blot analysis (Fig. 8A). The level of OCN, which is expressed at the final stage of osteogenesis, was high in the sequentially transfected hMSCs. The expression levels of these proteins were quantified (Fig. 8B). Similar to the Western blotting data, the OCN level was increased in sequentially transfected hMSCs. Immunohistological analysis of the various groups of hMSCs was performed (Fig. 8C). Red and green labeling, indicating COL1A2 and OCN proteins, respectively, was observed near to the masses of the hMSCs for 5 weeks of culture. Sequentially transfected hMSCs demonstrated vivid staining and many more spots (Fig. 8C,h,i,and p).

Although fluorescence representing COL1A2 and OCN was also detected in hMSCs transfected with DFs and with TFs, its intensity was lower than in sequentially transfected hMSCs (DFs/ATF4-14d) (Fig. 8C). This result indicates that sequential transfection of hMSCs can control the expression of specific ECM proteins and induce differentiation into mature osteoblasts. COL1A2 protein, an ECM marker of osteoblasts, was highly expressed in sequentially transfected hMSCs (Fig. S6). However, expression of COL2A1 protein, an ECM marker of chondrocytes, was lowest in sequentially transfected hMSCs (Fig. S6,n and o). This demonstrates that chondrogenesis of hMSCs was decreased, while osteogenesis of hMSCs was increased.

## Discussion

A combination of exogenous genes must be delivered to promote osteogenesis of stem cells. Genes expressed early in osteogenesis must be delivered first to trigger differentiation. Genes related to the final stage must be subsequently delivered to drive osteogenesis. In this study, we devised a powerful method to deliver specific target genes into stem cells using non-toxic nanoparticles (NPs). This method can easily control osteogenesis of stem cells. First, human mesenchymal stem cells (hMSCs) were treated with poly(lactic-co-glycolic acid) (PLGA) NPs coated with plasmid DNAs (pDNAs) harboring the RUNX2 and SP7 genes, involved in the early stage of osteogenesis. The pDNAs entered the nuclei of hMSCs, and RUNX2 and SP7 proteins were translated and triggered osteogenesis. Second, the ATF4 gene was delivered when cells were at the pre-osteoblast stage. ATF4 protein enhanced osteogenesis of stem cells.

Stem cell therapies have been met with some success in recent studies for the treatment of various human diseases. However, multipotent differentiation ability of stem cell has limited the utilization of this approach for cell therapy and tissue engineering protocols. One feasible strategy is to transfer genes that encode correlative differentiation into stem cell via safe and effective vehicles. In addition, the efficiency and safety tracking of the transplanted cells is crucial for distinguishing whether cellular regeneration originated from an exogenous cell source without post-mortem histology. To achieve this, various gene delivery methods for have been applied to differentiate the transplanted stem cells both *in vitro* and *in vivo*. However, stem cell differentiation is limited in terms of the translation of the transcript factor effects on stem cells. Here, we report an easy and versatile approach for obtaining new trials with a high transfection efficiency by gene delivery at certain time intervals. To avoid the simultaneous delivery of the genes, the sequential transfection of three different genes was performed through a facile surface modification process of PLGA nanoparticles. Here, PLGA nanoparticles as a drug carrier have been fabricated for the sequential gene transfection into hMSCs which then causes osteogenic differentiation. These results imply that the sequential gene transfection into hMSCs developed in this study could be widely applied as a transfection and tracking platform in stem cell therapy.

The transfection process of the hMSCs affected their osteogenic differentiation. Transfection of hMSCs with ATF4 alone, RUNX2 plus SP7 (DFs), and all three genes at the sametime (TFs) induced their osteogenic differentiation. However, this was insufficient for the gene ration osteoblasts. Transfection of ATF4 after RUNX2/SP7 induced differentiation of hMSCs intomature osteoblasts. By regulating the timing of key gene delivery, hMSCs can be easily differentiated into the desired mature cells.

## Method

### Materials

Biodegradable polymer PLGA (33,000 kDa) was purchased from Boehringer-Ingelheim (Petersburg, VA, USA). PEI (25 kDa, branched) was purchased from Polysciences (Warrington, PA, USA). Phosphate-buffered saline (PBS), fetal bovine serum (FBS), andalpha minimum essential medium were purchased from Life Technologies Corporation(Carlsbad, CA, USA).

### Preparation of dexamethasone-loaded PLGA nanoparticles (DNPsP)

DNPsP were prepared using a water-in-oil-in-water solvent evaporation technique. Briefly, 100 mg PLGA and 1 mg dexamethasone were emulsified with 20 mL methylenechloride and 1 mL methanol by sonication for 30 s (Bandelin Electronic, UW 70/HD 70; tip,MS 72/D; Berlin, Germany). The resulting double emulsion in a syringe was dropped into 50 mL of a 2% (w/v) aqueous polyvinyl alcohol solution and mechanically stirred for 2 h at 600 rpm. Residual methylene chloride was evaporated under a vacuum for 6 h.

### hMSC culture

Bone marrow-derived hMSCs (Lot# 8F3520) were obtained from Lonza Walkersville Inc. (PT-2501, Walkersville, MD, USA) and sub-cultured using a conventional method for 5–7 passages.

### Preparation of RUNX2, SP7, and ATF4 pDNA expression vectors

The vectors used in this study were fabricated by recombinant PCR methods and confirmed by nucleotide sequencing. RUNX2, SP7, and ATF4 cDNAs were obtained by RTPCR from cultured SW1353 cells. The vector harboring EGFP-tagged RUNX2 was obtained by ligating the human RUNX2 open reading frame into the multiple cloning site of pEGFPC1 (Clontech Laboratories, Inc., CA, USA). The vector harboring DsRed-tagged SP7 wasobtained by ligating the human SP7 open reading frame into the multiple cloning site of pDsRed-C1 (Clontech). The vector harboring EYFP-tagged ATF4 (pATF4) was obtained by ligating the human ATF4 open reading frame into the multiple cloning site of pEYFP-C1 (Clontech).

### *In vitro* transfection efficiency of pDNA-coated DNPsP

hMSCs (3 × 10^5^ cells/well) were seeded in a 6-well plate, cultured at 37 °C in 5% CO2, rinsed twice, and pre-incubated for 1 h with 2 mL of all free medium at 37 °C. To estimate the transfection efficiency, the hMSCs were incubated with DNPsP coated with RUNX2, SP7, or ATF4 pDNA for 6 h at 37 °C, washed three times with 1 mL PBS to remove any free complexes, suspended in PBS, and then further incubated for 24 h. Then, the hMSCs were harvested and analyzed using a flow cytometer (Guava Technologies) equipped with a488/554 nm excitation laser. The presented data are the mean fluorescent signals for 10,000 cells. For confocal microscopy, the cells were fixed with 4% paraformaldehyde, mounted inmounting medium (Dako Cytomation), and visualized using a confocal laser scanning microscope (LSM 880 Meta; Zeiss). Fluorescence was monitored in the EGFP/YFP (excitation, 488 nm; emission, 520 nm), RFP (excitation, 547 nm; emission, 575 nm), and DAPI (excitation, 358 nm; emission, 461 nm) channels. In addition, RT-PCR and Western blotting were performed.

### RT-PCR analysis following transfection of pDNA-coated DNPsP

hMSCs (3 × 105 cells/well) were seeded in a 6-well plate and cultured at 37 °C in 5% CO_2_. The hMSCs were incubated with DNPsP coated with RUNX2, SP7, or ATF4 pDNA at 37°C for 6 h, washed three times with 1 mL PBS to remove free complexes, suspended in PBS, and then further incubated for 36 h. To investigate the expression of downstream molecules, hMSCs were harvested and subjected to the RT-PCR analysis. Total RNA was extracted from the hMSCs using the TRIzol reagent (Invitrogen, Carlsbad, CA, USA) according to the manufacturer’s instructions. The primer sequences were as follows: ATF4, sense 5′-CTG ACC ACG TTG GAT GAC AC-3′ and antisense 5′-GGG CTC ATA CAG ATG CCT CT-3′; CTNNB1, sense 5′-TCA TGC GTTCTC CTC AGA TG-3′ and antisense 5′-AAT CCA CTG GTG AAC CAA GC-3′; osteopontin (OPN), sense 5′-CAT CTC AGA AGC AGA ATC TC-3′ and antisense 5′-CCATAA ACC ACA CTA TCA CC-3′; osteocalcin (OCN), sense 5′-CCA GGC GCT ACC TGTATC AA-3′ and antisense 5′- AGG GGA AGA GGA AAG AAG GG-3′; and glyceraldehyde3-phosphate dehydrogenase (GAPDH), sense 5′-CGC TGA GTA CGT CGT GGA GT-3′ and antisense 5′-ATG ATG TTC TGG AGA GCC CC-3′. Reverse transcription was performed at 42 °C for 60 min using 500 ng total RNA. The PCR conditions for human ATF4 and GAPDH were as follows: 26 cycles of denaturation at 94 °C for 20 s, annealing at 61 °C for 30 s, and extension at 72 °C for 45 s, followed by the final extension at 70 °C for 7 min.

### Studies of osteogenesis according to the timing of pATF4-coated DNPsP transfection

The hM2SCs (3 × 10^5^ cells/well) were seeded in a 6-well plate and cultured at 37 °C in 5% CO2. The hMSCs were then transfected with pATF4-coated DNPsP at 5, 7, or 14 days after the transfection of the DNPsP coated with pDNAs and harboring RUNX2 and SP7. To determine the expression of differentiation markers according to the timing of pATF4 transfection, the cells were harvested 3 weeks after the first transfection and subjected to RT-PCR, Western blot, histological (von Kossa and alkaline phosphatase (ALP) staining), and immunological analyses. In addition, ALP and OCN enzyme-linked immunosorbent assays (ELISAs) were performed to compare bone calcification/mineralization according to the timing of the pATF4 transfection. ALP staining was performed using an ALP kit from Sigma (86 R; St. Louis, MO, USA) according to the manufacturer’s instructions. The cells were fixed in citrate-acetone-formaldehyde for 45 sec at room temperature prior to staining. The ALP activity was determined using an ALP assay kit (ab83369; Abcam, Cambridge, USA). A standard curve was created using p-nitrophenol, and each value was normalized to the protein concentration. ALP activity in each sample was normalized by the protein concentration and determined by measuring the absorbance at 405 nm using an ELISA reader. OCN was quantified using an OCN Human Simple Step ELISA kit from Abcam (ab195214; Cambridge, USA). The wells were pre-coated with a mouse monoclonal antibody against OCN for 2 h at room temperature. After three washes with buffer, 200 μL of an antimouseh or seradish peroxidase-conjugated polyclonal antibody was added to each well for 2 hat room temperature. After further washing, 200 μL of a solution containing hydrogenperoxide and TMB chromogen at the ratio of 1:1 was added to each well for 20 min. Thereafter, 50 μL Stop Solution was added to each well and absorbance at 450 nm was measured within 30 min, with the wave length correction set to 540 or 570 nm.The quantitative data (e.g. ELISAs) measurements were repeated 5 times in this study.

### Three-dimensional (3D) culture

To prepare each pellet, 1 × 10^6^ cells in 1 mL defined medium were centrifuged at 1,200 rpm for 3 min in a 15 mL conical tube. The pellets were divided into three parts in complete medium (containing 10% FBS and 1% antibiotics) and cultured at 37 °C in 5% CO2 for 5 weeks, changing the medium every 2–3 days.

### Evaluation of osteogenesis upon pDNA-coated DNPsP delivery in 3D cultures

To investigate bone formation, the pellets were harvested and subjected to real-time PCR, Western blot, histological (von Kossa and Alizarin Red S staining), and immunological analyses. For histology, the samples were placed in the optimum cutting temperature material (TISSUETEK4583; Sakura Finetek USA, Inc.) for freezing. The frozen samples were sliced intosections (5–10 μm thick) at −20 °C and stained with von Kossa and Alizarin Red S. Immunofluorescence analyses were conducted to identify OCN (Abcam, Cambridge, UK), collagen type I (COL1A2; Millipore, Temecula, CA, USA), and ALP (Abcam, Cambridge, UK) by incubation with specific antibodies in humidified conditions. The samples were then stained with fluorescently labeled secondary antibodies (1:500; Thermo Scientific, PT, USA). Following three rinses with PBS, the sections were incubated with DAPI (1:1000) for 2 min and mounted in an aqueous/dry mounting medium (Dako).

### Statistical analysis

The Student’s t-test was used for all statistical analyses. *P < 0.05 and **P < 0.01 were considered to be statistically significant.

## Electronic supplementary material

Supplementary Information
