# Author Correction: Sequential transfection of RUNX2/SP7 and ATF4 coated onto dexamethasone-loaded nanospheres enhances osteogenesis

**DOI:** 10.1038/s41598-019-45277-x

**Published:** 2019-06-26

**Authors:** Hye Jin Kim, Ji Sun Park, Se Won Yi, Hyun Jyung Oh, Jae-Hwan Kim, Keun-Hong Park

**Affiliations:** Department of Biomedical Science, College of Life Science, CHA University, 6F, CHA Biocomplex, 689 Sampyeongdong Bundang-gu, Seongnam-si, 134-88 Korea

Correction to: *Scientific Reports* 10.1038/s41598-018-19824-x, published online 23 January 2017

The original version of this Article contained an error in the title of the paper, where “nanospheres enhances” was incorrectly given as “nanospheresenhances”.

In addition, this Article contained an error in the Acknowledgements section,

“and the Next-Generation BioGreen 21 Program (No.PJ000000), Rural Development Administration, Republic of Korea.”

now reads:

“and the Next-Generation BioGreen 21 Program (No.PJ01122202), Rural Development Administration, Republic of Korea.”

These errors have now been corrected in the PDF and HTML versions of the Article.

