# Supplementary material for: Sequential transfection of RUNX2/SP7 and ATF4 coated onto dexamethasone-loaded nanospheres enhances osteogenesis
Source: Sci Rep. 2018 Jan 23;8:1447. doi: 10.1038/s41598-018-19824-x (PMC5780485; doi:10.1038/s41598-018-19824-x)
Supplement: Supplementary file 1 — Supplementary Information [file 41598_2018_19824_MOESM1_ESM.pdf]

Scientific Reports

Supplementary Information

Sequential transfection of Runx2/Sp7 and ATF4 Coated onto dexamethasone loaded nanospheres enhances osteogenesis

Hye Jin Kim, Ji Sun Park, Se Won Yi, Hyun Jyung Oh, Jae-Hwan Kim*, Keun-Hong Park*

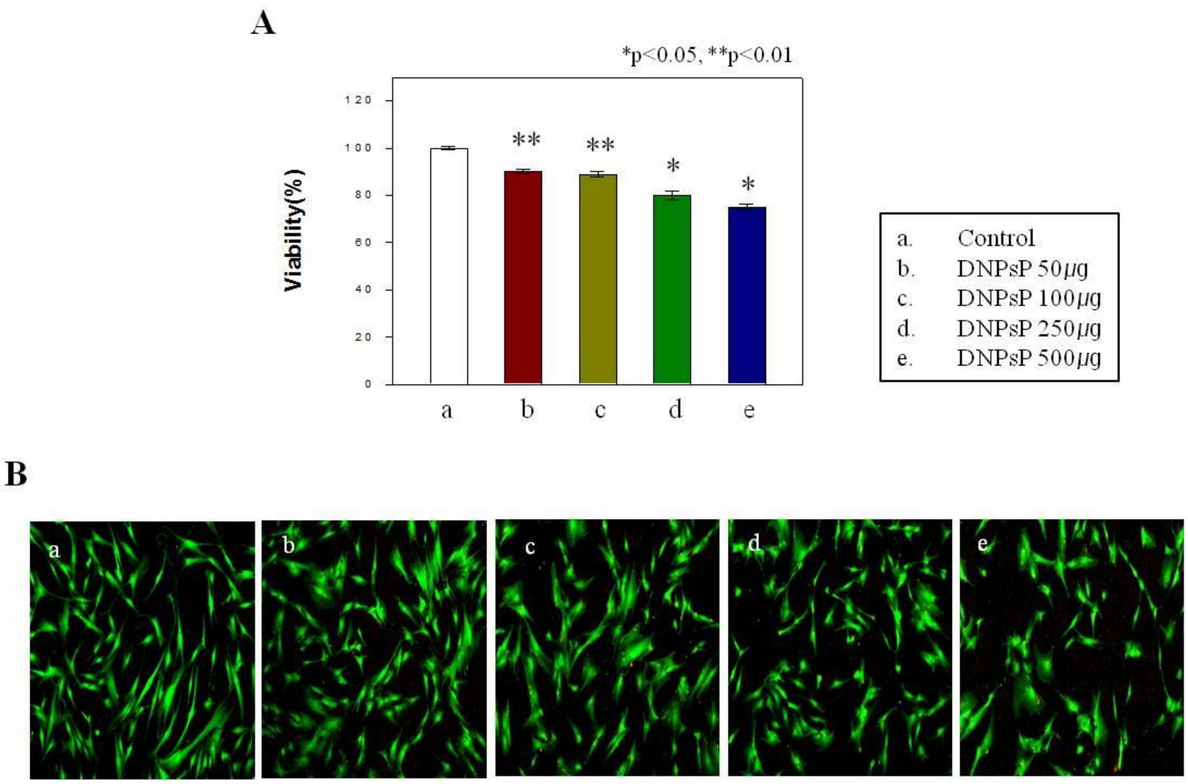

Figure S1.In vitro viability of hMSCs. Related to Figure 1.

A:CCK-8 assay of (a) control hMSCs and those treated with (b) DNPsP (50 µg), (c) DNPsP (100 µg), (d) DNPsP (250 µg), and DNPsP (500 µg) for 6 h.

B: Live/dead imaging assay of (a) control hMSCs and those treated with (b) DNPsP (50 µg), (c) DNPsP (100 µg), (d) DNPsP (250 µg), and (e) DNPsP (500 µg) for 6 h.

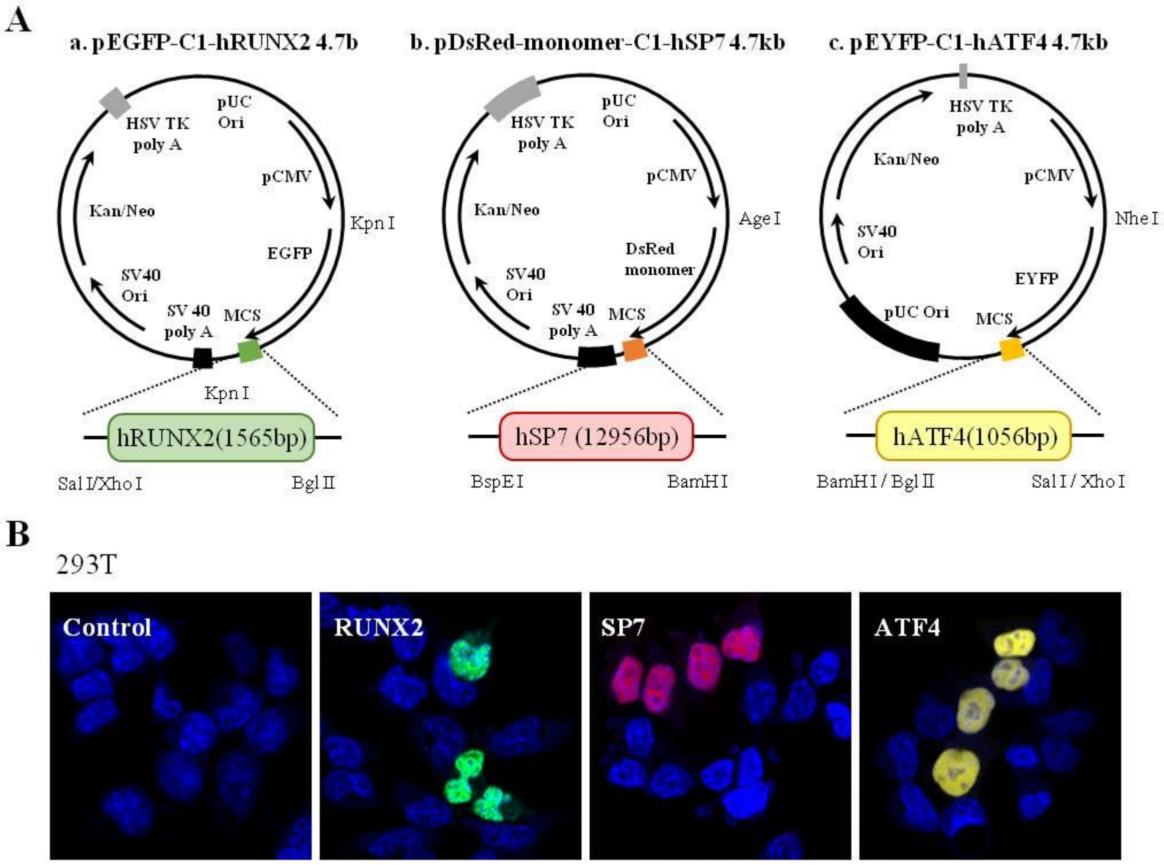

Figure S2.Fabrication and characterization of pDNAs harboring the RUNX2, Sp7, and ATF4 genes. Related to Figure 1.

A: Maps of plasmids harboring (a) EGFP-RUNX2, (b) dsRed-Sp7, and (c) EYFP-ATF4.

B: Transfection of 293T cells with the plasmids was detected by confocal laser microscopy.

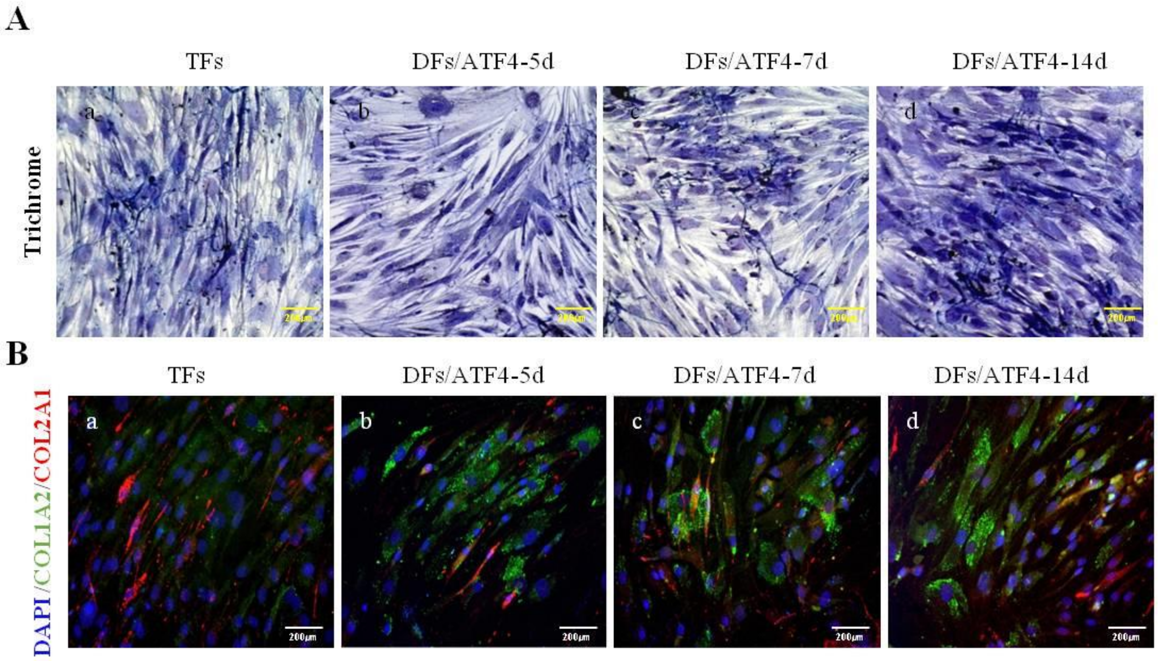

Figure S3.Histological analysis of hMSCs transfected with ATF4 at 5, 7, and 14 days after RUNX2/Sp7 in a 2D culture system. Related to Figure 4.

A: Masson's Trichrome staining of hMSCs transfected with (a) all three genes at the same time point or with pATF4 at (b) 5 days, (c) 7 days, and (d) 14 days after RUNX2/Sp7. Scale bar, 100 μm.

B: Immunofluorescence of hMSCs transfected with (a) all three genes at the same time point or with ATF4 at (b) 5 days, (c) 7 days, and (d) 14 days after RUNX2/Sp7 (blue, DAPI; green, COLI; and red, COLII). Scale bar, 100 μm.

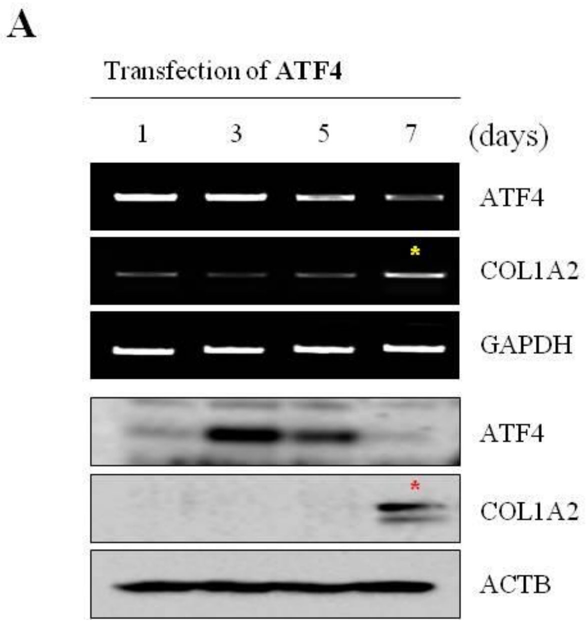

Figure S4.Genes expressed in hMSCs transfected with ATF4. Related to Figure 5.

A: RT-PCR and Western blot analyses of mRNA and protein expression levels of ATF4 and COLI in hMSCs transfected with ATF4 at 14 days after RUNX2/Sp7.

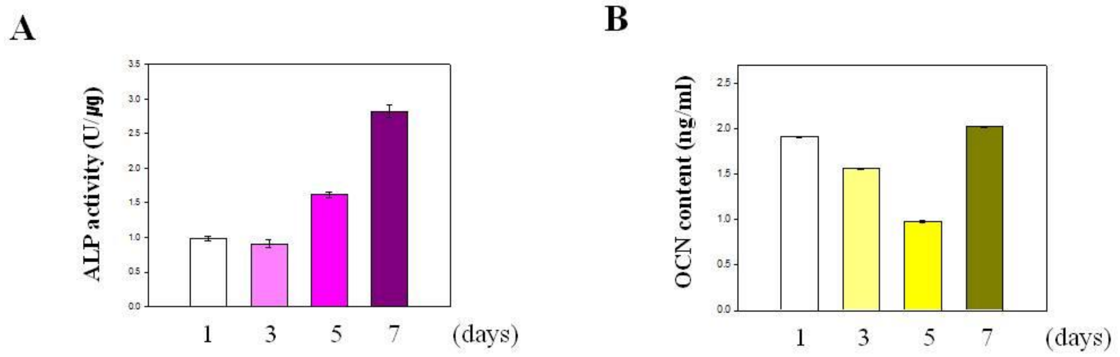

Figure S5.Osteogenesis of hMSCs transfected with ATF4 at 14 days after RUNX2/Sp7 and cultured for various durations. Related to Figure 5.

A: ALP activity in hMSCs transfected with ATF4 at 14 days after RUNX2/Sp7 and cultured for 1, 3, 5, and 7 days.

B:OCN production in hMSCs transfected with ATF4 at 14 days after RUNX2/Sp7 and cultured for 1, 3, 5, and 7 days (mean ± SD of three experiments; ∗, $P < 0.01$).

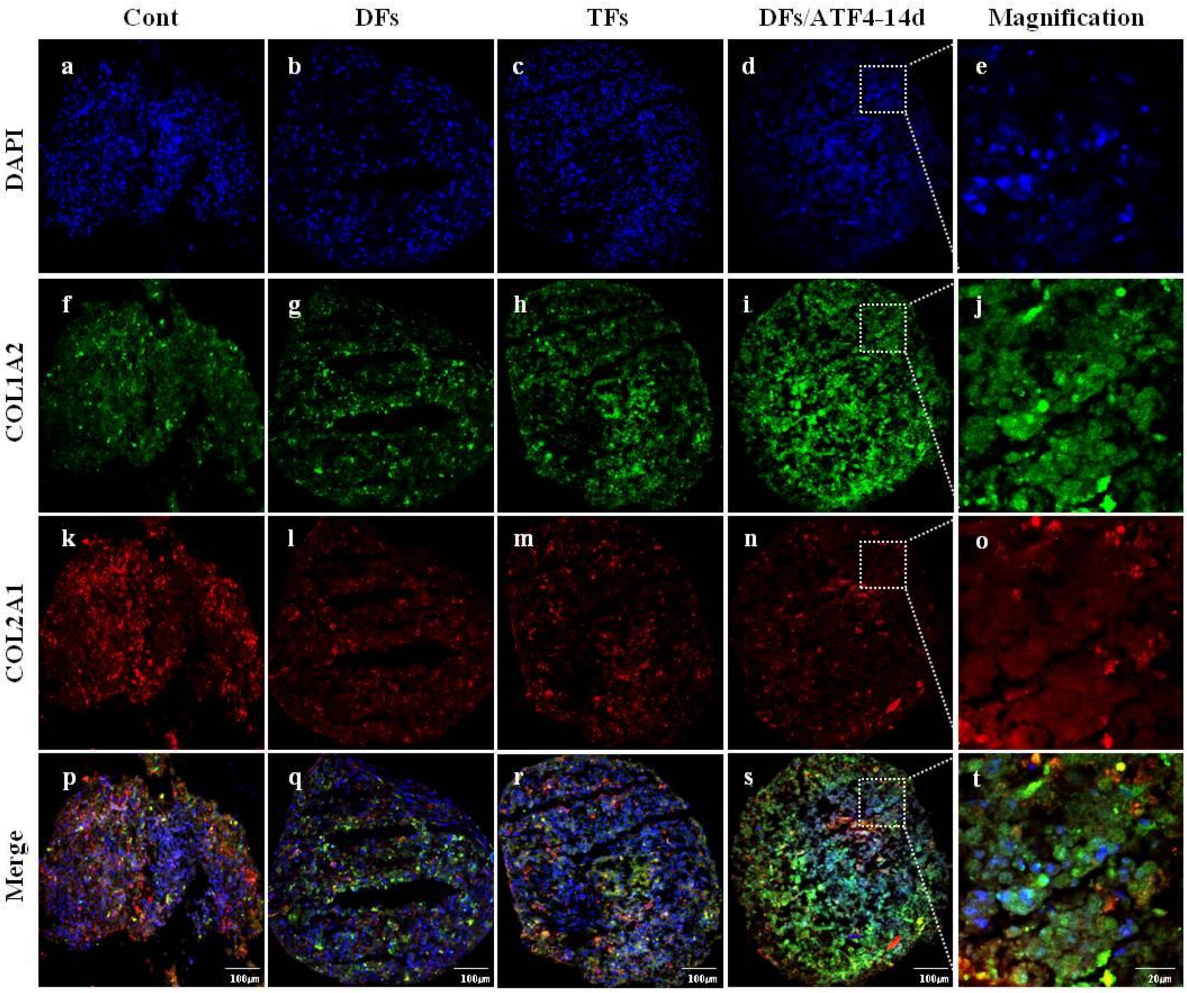

Figure S6.Osteogenesis of (a, f, k, and p) control hMSCs and those transfected with (b, g, l, and q) RUNX2 plus Sp7, (c, h, m, and r) all three genes at the same time point, and (d, i, n, and s) ATF4 at 14 days after RUNX2/Sp7 in a 3D culture system. Related to Figure 8.

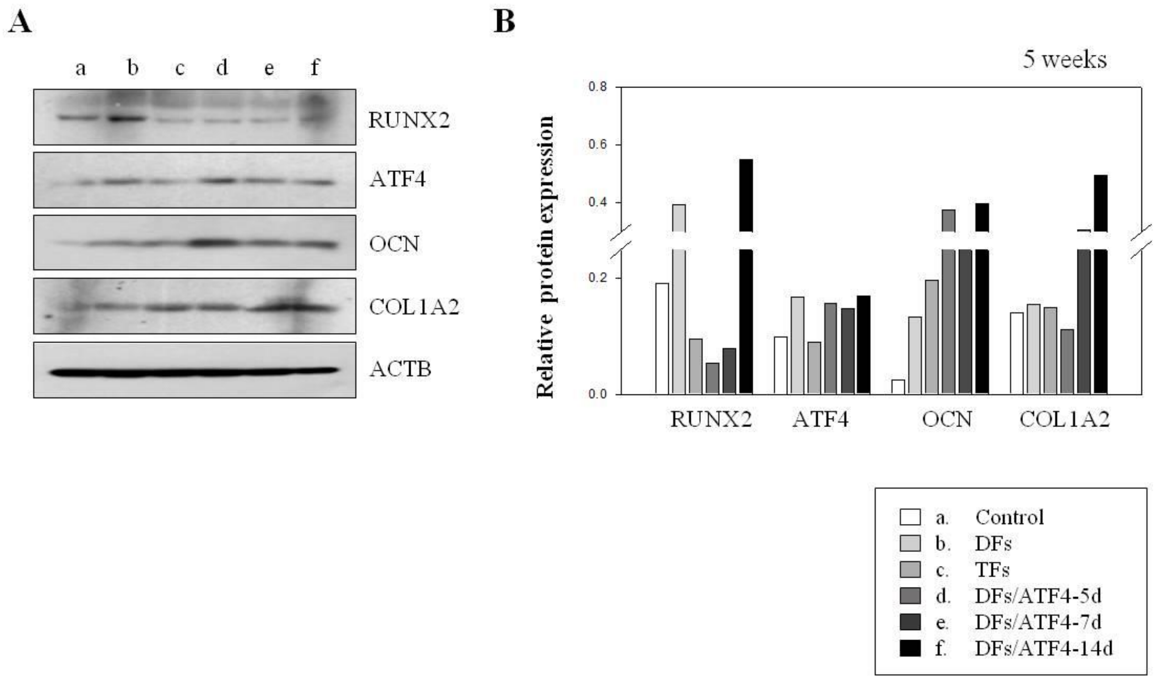

Figure S7.Osteogenesis of control hMSCs and those transfected with two and three genes in a 3D culture system. Related to Figure 8.

A: Protein expression levels of RUNX2, ATF4, OCN, and COLI in (a) control hMSCs and those transfected with (b) two genes, (c) all three genes at the same point, and ATF4 at (d) 5 days, (e) 7 days, and (f) 14 days after RUNX2/Sp7.

B: Quantification of the protein levels of RUNX2, ATF4, OCN, and COLI in (a) control hMSCs and those transfected with (b) two genes, (c) all three genes at the same time point, and ATF4 at (d) 5 days, (e) 7 days, and (f) 14 days after RUNX2/Sp7.

SUPPLEMENTAL EXPERIMENTAL PRODURES

Preparation of RUNX2, Sp7, and ATF4 pDNA expression vectors

The vectors used in this study were fabricated by recombinant PCR methods andconfirmed by nucleotide sequencing. RUNX2, Sp7, and ATF4 cDNAs were obtained by RTPCRfrom cultured SW1353 cells. The vector harboring EGFP-tagged RUNX2 was obtainedby ligating the human RUNX2 open reading frame into the multiple cloning site of pEGFPC1(Clontech Laboratories, Inc., CA, USA). The vector harboring DsRed-tagged SP7 wasobtained by ligating the human Sp7 open reading frame into the multiple cloning site ofpDsRed-C1 (Clontech). The vector harboring EYFP-tagged ATF4 (pATF4) was obtained byligating the human ATF4 open reading frame into the multiple cloning site of pEYFP-C1 (Clontech).

In vitro transfection efficiency of pDNA-coated DNPsP

hMSCs (3 × 10^5 cells/well) were seeded in a 6-well plate, cultured at 37°C in 5% CO2,rinsed twice, and pre-incubated for 1 h with 2 mL of all free medium at 37°C. To estimate thetransfection efficiency, hMSCs were incubated with DNPsP coated with RUNX2, Sp7, orATF4 pDNA for 6 h at 37°C, washed three times with 1 mL PBS to remove any freecomplexes, suspended in PBS, and further incubated for 24 h. Thereafter, hMSCs wereharvested and analyzed using a flow cytometer (Guava Technologies) equipped with a488/554 nm excitation laser. The data shown are the mean fluorescent signals for 10,000cells. For confocal microscopy, cells were fixed with 4% paraformaldehyde, mounted inmounting medium (Dako Cytomation), and visualized using a confocal laser scanningmicroscope (LSM 880 Meta; Zeiss). Fluorescence was monitored in the EGFP/YFP(excitation, 488 nm; emission, 520 nm), RFP (excitation, 547 nm; emission, 575 nm), andDAPI (excitation, 358 nm; emission, 461 nm) channels. In addition, RT-PCR and Westernblotting were performed.

RT-PCR analysis following transfection of pDNA-coated DNPsP

hMSCs (3 × 105 cells/well) were seeded in a 6-well plate and cultured at 37°C in 5%CO2. hMSCs were incubated with DNPsP coated with RUNX2, Sp7, or ATF4 pDNA at 37°Cfor 6 h, washed three times with 1 mL PBS to remove free complexes, suspended in PBS, andfurther incubated for 36 h.To investigate expression of downstream molecules, hMSCs were harvested andsubjected to RT-PCR analysis. Total RNA was extracted from hMSCs using TRIzol reagent(Invitrogen, Carlsbad, CA, USA) according to the manufacturer’s instructions. The primersequences were as follows: ATF4, sense 5′-CTG ACC ACG TTG GAT GAC AC-3′ andantisense 5′-

GGG CTC ATA CAG ATG CCT CT-3′; β-catenin, sense 5′-TCA TGC GTTCTC CTC AGA TG-3′ and antisense 5′-AAT CCA CTG GTG AAC CAA GC-3′;osteopontin (OPN), sense 5'-CAT CTC AGA AGC AGA ATC TC-3' and antisense 5'-CCATAA ACC ACA CTA TCA CC-3'; osteocalcin (OCN), sense 5'-CCA GGC GCT ACC TGTATC AA-3' and antisense 5'- AGG GGA AGA GGA AAG AAG GG-3'; and glyceraldehyde3-phosphate dehydrogenase (GAPDH), sense 5′-CGC TGA GTA CGT CGT GGA GT-3′ andantisense 5′-ATG ATG TTC TGG AGA GCC CC-3′. Reverse transcription was performed at42°C for 60 min using 500 ng total RNA. The PCR conditions for human ATF4 and GAPDHwere as follows: 26 cycles of denaturation at 94°C for 20 s, annealing at 61°C for 30 s, andextension at 72°C for 45 s, followed by a final extension at 70°C for 7 min.

Studies of osteogenesis according to the timing of pATF4-coated DNPsPtransfection

hM2SCs (3 × 105 cells/well) were seeded in a 6-well plate and cultured at 37°C in 5%

CO_2. hMSCs were transfected with pATF4-coated DNPsP at 5, 7, or 14 days aftertransfection of DNPsP coated with pDNAs harboring RUNX2 and Sp7. To determine theexpression of differentiation markers according to the timing of pATF4 transfection, cellswere harvested 3 weeks after the first transfection and subjected to RT-PCR, Western blot,histological (von Kossa and alkaline phosphatase (ALP) staining), and immunologicalanalyses. In addition, ALP and OCN enzyme-linked immunosorbent assays (ELISAs) wereperformed to compare bone calcification/mineralization according to the timing of pATF4transfection.ALP staining was performed using an ALP kit from Sigma (86R; St. Louis, MO, USA)according to the manufacturer’s instructions. Cells were fixed in citrate-acetoneformaldehydefor 45 sec at room temperature before staining. ALP activity was determinedusing an ALP assay kit (ab83369; Abcam, Cambridge, USA). A standard curve was createdusing p-nitrophenol, and each value was normalized to the protein concentration. ALPactivity in each sample was normalized by the protein concentration and determined bymeasuring absorbance at 405 nm using an ELISA reader.OCN was quantified using an OCN Human Simple Step ELISA kit from Abcam(ab195214; Cambridge, USA). Wells were pre-coated with a mouse monoclonal antibodyagainst OCN for 2 h at room temperature. After three washes with buffer, 200 µL of an antimousehorseradish peroxidase-conjugated polyclonal antibody was added to each well for 2 hat room temperature. After further washing, 200 µL of a solution containing hydrogenperoxide and TMB chromogen at a ratio of 1:1 was added to each well for 20 min.Thereafter, 50 µL Stop Solution was added to each well and absorbance at 450 nm wasmeasured within 30 min, with the wavelength correction set to 540 or 570 nm.

Three-dimensional (3D) culture

To prepare each pellet, 1 × 10^6 cells in 1 mL defined medium were centrifuged at 1,200rpm for 3 min in a 15 mL conical tube. Pellets were divided into three in complete medium(containing 10% FBS and 1% antibiotics) and cultured at 37°C in 5% CO2 for 5 weeks,changing the medium every 2–3 days.

Evaluation of osteogenesis upon pDNA-coated DNPsP delivery in 3D cultures

To investigate bone formation, pellets were harvested and subjected to real-time PCR,Western blot, histological (von Kossa and Alizarin Red S staining), and immunologicalanalyses.For histology, samples were placed in optimum cutting temperature material (TISSUETEK4583; Sakura Finetek USA, Inc.) for freezing. The frozen samples were sliced intosections (5–10 μm thick) at -20°C and stained with von Kossa and Alizarin Red S.Immunofluorescence analyses were conducted to identify OCN (Abcam, Cambridge,UK), collagen type I (COLI; Millipore, Temecula, CA, USA), and ALP (Abcam, Cambridge,UK) by incubation with specific antibodies in humidified conditions. Samples were thenstained with fluorescently labeled secondary antibodies (1:500; Thermo Scientific, PT, USA).Following three rinses with PBS, sections were incubated with DAPI (1:1000) for 2 min andmounted in aqueous/dry mounting medium (Dako).

Statistical analysis

The Student's t-test was used for all statistical analyses. *P<0.05 and **P<0.01 wereconsidered to be statistically significant.
